# Serendipity; Close Encounter of Tenascin C

**DOI:** 10.3389/fimmu.2020.620182

**Published:** 2021-02-03

**Authors:** Teruyo Sakakura

**Affiliations:** Department of Matrix Biology and Pathology, Mie University Graduate School of Medicine, Tsu City, Japan

**Keywords:** epithelial-mesenchymal interactions, morphogenesis, mammary gland, cancer, stroma

## Introduction

In 1924, Hans Spemann and Hilde Mangold established the concept of embryonic induction (1). They transplanted the dorsal grey crescent region of one newt gastrula to the ventral part of another newt gastrula and found that a piece of the transplant induced the host tissue into the formation of a secondary embryo. With this as a starting point, the interactions among various tissues became the subject of follow up research during the next decade. In the 1950s, Clifford Grobstein discovered that organogenesis of glandular tissues in prenatal mice required morphogenetic interactions between epithelium and mesenchyme (2). Consequently, epithelial-mesenchymal interactions became a hot topic for developmental biologists worldwide. From 1967 to 1981, similar experiments were done on various organs such as salivary glands, kidney, pancreas, digestive tract, prostate, tooth, lung and mammary gland, etc.

In 1969, Klaus Kratochwil observed typical mammary gland morphogenesis by monopodial branching pattern in recombinant cultures of embryonic mammary epithelium with mammary mesenchyme. By contrast, mammary epithelium cultured in contact with salivary gland mesenchyme showed salivary gland like morphogenesis by dichotomous branching pattern (3). Prompted by this observation, Teruyo Sakakura and her colleagues conducted further analysis using *in vivo* system in 1976. They transplanted embryonic mammary epithelium combined with mammary or salivary mesenchyme under the kidney capsule and made the host female mice pregnant and lactating. The results were clear cut. Both grafted tissues produced milk (4). That is, mammary gland morphogenesis is mesenchyme-dependent, and cytodifferentiation is epithelium-specific. The ability to recapitulate morphogenesis persists in adult mammary epithelium. When embryonic mammary or salivary mesenchyme is transplanted into adult mammary gland, the epithelial cells in contact with the mesenchymal graft proliferate in multiple layers, forming duct-alveolar hyperplasia with nodular structure rapidly becoming cancerous (5, 6). Based upon these results, she built up a hypothesis that embryonic changes in the stroma must occur during cancer development.

## Discovery of Tenascin as an Oncofetal ECM Protein

In 1984, Sakakura was invited by the director Edward Reich of the Friedrich-Miescher Institute in Basel asking her to organize a new group of breast cancer research. Ruth Chiquet-Ehrismann (Swiss), Eleanor J Mackie (Australian) and Caroline A Pearson (American) were the members of the new group. The research project was to identify stromal markers common in embryonic and malignant mammary glands. Extracellular matrix (ECM) proteins were hopeful, because the interactions between epithelium and mesenchyme are not by diffusible factors but by matrix-bound morphogens. They had collected various antibodies against ECM substances. Sprague-Dawley rats were used in the experiments. Mammary tumors were induced by intravenous injection of N-methyl-N-nitrosourea. By fluorescent antibody method they stained embryonic mammary glands and cancers. Among them, an antibody against an ECM protein already reported as “myotendinous antigen” (7) was found to be suitable molecule and the molecule was renamed “tenascin”. Ruth and her husband Matthias Chiquet (Univresity of Basel) were the godparents. Thus, the discovery of tenascin-C as an oncofetal ECM protein was indeed by serendipity. The photographs of mammary tumors stained with anti-tenascin antibody were chosen as the cover of the Cell journal (8). As for the function of tenascin, growth stimulation of tumor cells was suggested. When rat mammary tumor cells were cultured on the plates coated with several ECM proteins (tenascin, fibronectin, collagen I, collagen IV, and laminin), tenascin was a poor adhesive substrate but nevertheless the most effective in promoting cell growth after serum was removed from the culture medium (8).

In the period of 1983 to 1985, several other ECM glycoproteins had been reported independently from different laboratories: glioma-mesenchymal extracellular matrix (GMEM) (9), hexabrachion (10), J1 (11), and cytotactin (12). In terms of their gene structures and electron microscopy images, all of these molecules were considered to be identical to tenascin. The protein was renamed tenascin C (TN-C) later.

After Sakakura returned to Japan, she organized several groups at the Aichi Cancer Center Research Institute, then RIKEN and Mie University School of Medicine. These groups carried out establishment of the purification method of human TN-C and production of monoclonal antibodies against human TN-C (13), cDNA cloning of mouse TN-C (14), and generation of lines of TN-C knockout mice (15). TN-C knockout mice were initially found to be viable and fertile, but that further studies clearly showed the importance of TN-C as a disease-associated stress protein later in life.

We happened to meet tenascin by sheer serendipity. TN-C is always detected in the stroma when the tissue is under reconstruction, such as developmental morphogenesis, inflammatory process, cancer cell growth and invasion. It is just like an ambulance to appear in an accident.

## Author Contributions

The author confirms being the sole contributor of this work and has approved it for publication.

## Conflict of Interest

The author declares that the research was conducted in the absence of any commercial or financial relationships that could be construed as a potential conflict of interest.

## References

[1] Spemann H Mangold H Induction of embryonic primordia by implantation on organizers from a different species. Int J Dev Biol (1924) 45:13–38. 11291841

[2] Grobstein C Morphogenetic interaction between embryonic mouse tissues separated by membrane filter. Nature (1953) 172:869–71. 10.1038/172869a0 13111219

[3] Kratochwil K Organ specificity in mesenchymal induction demonstrated in the embryonic development of the mammary gland of the mouse. Dev Biol (1969) 20:46–71. 10.1016/0012-1606(69)90004-9 5795848

[4] Sakakura T Nishizuka Y Dawe CJ Mesenchyme-dependent morphogenesis and epithelium-specific cytodifferentiation in mouse mammary gland. Science. (1976) 194:1439–41. 10.126/science.827022 827022

[5] Sakakura T Sakagami Y Nishizuka Y Persistence of responsiveness of adult mouse mammary gland to induction by embryonic mesenchyme. Dev Biol (1979) 72:201–19. 10.1016/0012-1606(79)90111-8 510783

[6] Sakakura T Sakagami Y Nishizuka Y Accelerated mammary cancer development by fetal salivary mesenchyme isografted to adult mammary epithelium. J Natl Cancer Inst (1981) 66:953–9. 6262561

[7] Chiquet M Fambrough DM Chick myotendinous antigen. I. A monoclonal antibody as a marker for tendon and muscle morphogeneis. J Cell Biol (1984) 98:1926–36. 10.083/jcb.98.6 PMC2113040 6725406

[8] Chiquet-Ehrismann R Mackie EJ Pearson CA Sakakura T Tenascin: an extracellular matrix protein involved in tissue interactions during fetal development and oncogenesis. Cell. (1986) 47:131–9. 10.1016/0092-8674(86)90374-0 2428505

[9] Bourdon MA Wikstrand CJ Fruthmayr H Matthews TJ Bigner DD Human glioma-mesenchymal extracellular matrix antigen defined by monoclonal antibody. Cancer Res (1983) 43:2796–805. 6342760

[10] Erickson HP Inglesias JL A six-armed oligomer isolated from cell surface fibronectin preparations. Nature (1984) 311:267–9. 10.1038/311267a0 6482952

[11] Kruse J Keihauer G Faissner A Timple R Schachner M The J1 glycoprotein : A novel nervous system cell adhesion molecule of the L2/HNK-1 family. Nature. (1985) 316:146–8. 10.1038/316146a0 2409452

[12] Grumet M Hoffman S Grossin KL Edelman GM Cytotactin, and extracellular matrix protein of neural and non-neural tissues that mediates glia-neuron interaction. Proc Natl Acad Sci USA. (1985) 82:8075–9. 10.1073/pnas.82.23.8075 PMC391445 2415980

[13] Oike Y Hiraiwa N Kawakatsu H Nishikai M Okinaka T Suzuki T Isolation and characterization of human fibfroblast tenascin. An extracellular matrix plycoprotein of interest for developmental studies. Int J Dev Biol (1990) 34:309–17. 1696829

[14] Saga Y Tsukamoto T Jing N Kusakabe M Sakakura T Mouse tenascin: cDNA cloning and spatiotemporal expression of isoforms. Gene. (1991) 104:177–85. 10.1016/0378-1119(91)90248-a 1717349

[15] Saga Y Yagi T Ikawa Y Sakakura T Aizawa S Mice develop normally without tenascin. Genes Dev (1992) 6:1821–31. 10.101/gad.6.10 1383086

